# Advances in Understanding and Treating Amyotrophic Lateral Sclerosis (ALS): A Comprehensive Review

**DOI:** 10.7759/cureus.48691

**Published:** 2023-11-12

**Authors:** Deepal Gupta, Sunita Vagha, Hitaansh Dhingra, Hrishita Shirsath

**Affiliations:** 1 Medicine, Jawaharlal Nehru Medical College, Datta Meghe Institute of Higher Education and Research, Wardha, IND; 2 Pathology, Jawaharlal Nehru Medical College, Datta Meghe Institute of Higher Education and Research, Wardha, IND

**Keywords:** physical medicine and rehabilitation, stem cell therapy, neurodegenerative disesase, masitinib, human genetics and epigenetics, familial amyotrophic lateral sclerosis

## Abstract

Amyotrophic lateral sclerosis (ALS) is a deadly CNS neurodegenerative disease. The way ALS is now managed, from diagnosis to prognosis, is still not ideal despite many studies. Early diagnosis can help ALS patients live longer since prompt treatment can halt the disease's development. Two medications, riluzole and edaravone, have recently been licensed for use in therapy, and they very slightly increase life expectancy. Still, a lot of cutting-edge experimental medications are being developed. In the following article, we give a synopsis of the innovative medications and genetic remodeling that have emerged recently and help to halt the course of the illness. Studies have also been conducted on a few symptomatic and rehabilitative therapies that enhance the quality of life for ALS patients.

## Introduction and background

Both upper and lower motor neurons (MNs) can be affected by the debilitating neurodegenerative illness amyotrophic lateral sclerosis (ALS), which has a bad prognosis. Only four medications, riluzole, dextromethorphan hydrobromide with quinidine sulfate, edaravone, and sodium phenylbutyrate with taurursodiol, were approved by the FDA to treat ALS between 1995 and October 2022. Even with the usage of these four authorized medications, ALS patients still have poor quality of life and a low survival probability. Therefore, it is imperative to identify new therapies for ALS patients [1]. Fifteen percent of patients have a family history of frontotemporal dementia (FTD) or ALS, while the remaining cases appear to be sporadic in nature. The disease's pathological features include the loss of both upper and lower motor neurons as well as a selective degeneration of motor pathways. The accumulation of the protein TDP43 in the cytoplasm of impacted neurons, seen in almost 97% of instances (patients with SOD1 and FUSgene mutations being the only exceptions), is a defining feature of the disease process. The onset of ALS typically occurs between 40 and 70 years of age, with sporadic forms highest at 58-63 years and familial forms peaking at 47-52 years [2,3]. Patients are identified based on both a diaphragmatic pattern of respiratory weakness and an asymmetric, subtle development of distal painless weakness or muscle atrophy. Respiratory failure is the primary cause of mortality, and it usually happens three to five years after diagnosis [4,5].

## Review

Methodology

Literature Search

We used a variety of databases, including Google Scholar and PubMed. Particular terms pertinent to our research were included in our search, such as "Amyotrophic lateral sclerosis", "treatment", "newer drugs", "epigenetics" and "pharmacotherapy". In order to find more research, we also manually examined the relevant papers' reference lists.

Inclusion and Exclusion Criteria

Our study included investigations on the pathophysiology, clinical characteristics, management, and alleviation of symptoms related to ALS, articles ranging from publishing years 2012 to 2022. However, we decided to exclude research articles that were not in English and studies that required payment to view.

Data Extraction and Synthesis

We collected information from each study that was a component of the research in order to conduct our analysis. We used a narrative method to analyze this material efficiently, allowing us to emphasize pertinent and up-to-date aspects while offering an overview.

Data Analysis

**Figure 1:**
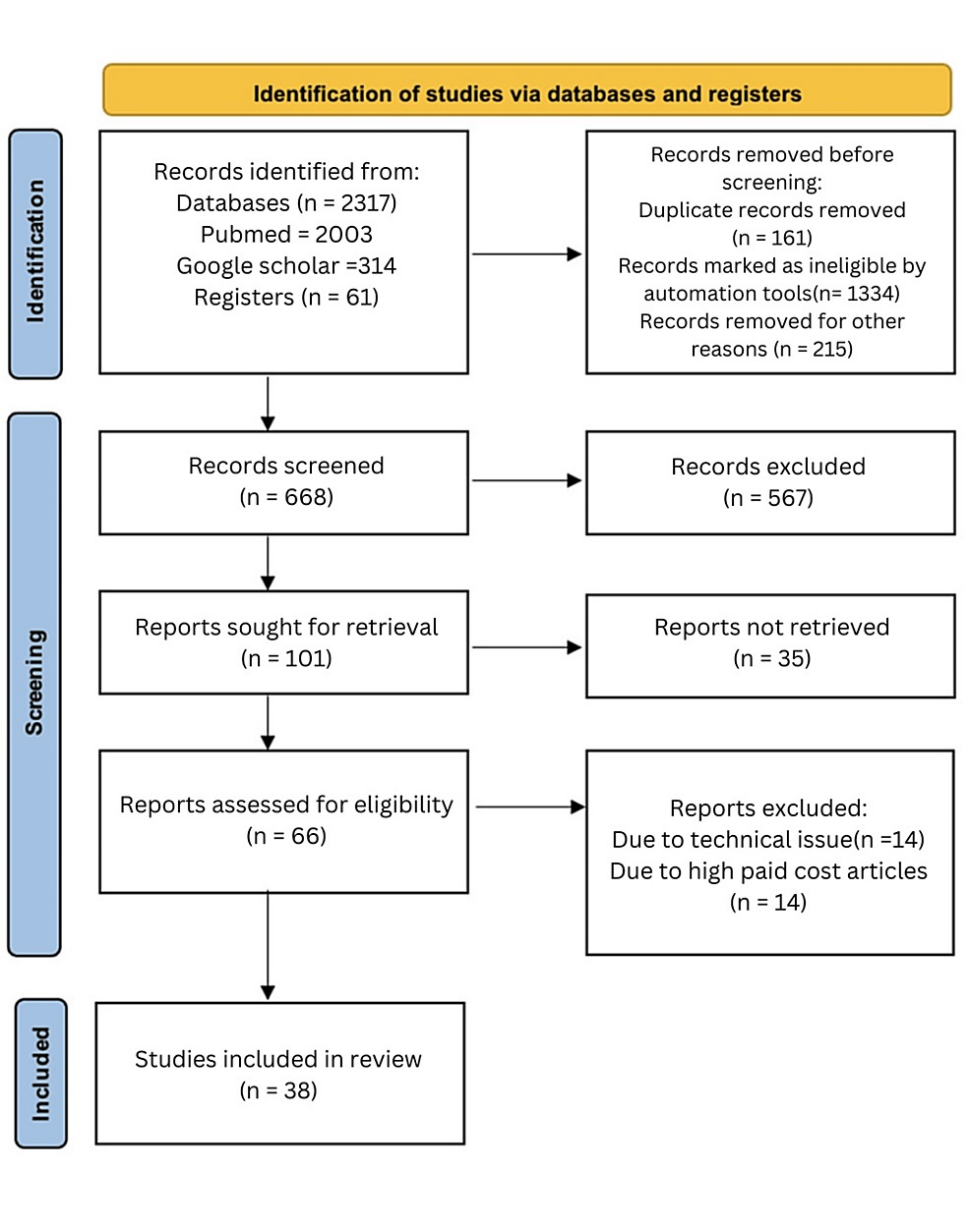
Search strategy using the PRISMA method PRISMA: Preferred Reporting Items for Systemic Review and Meta-Analyses

We looked at the data using a qualitative approach in our analysis. Finding recurring themes and patterns in the papers that made up our research was our main goal. The employed search technique is depicted in Figure 1.

Disease pathology 

Like other neurodegenerative diseases, ALS is believed to be brought on by an interaction of aging-related malfunction, environmental variables, and hereditary factors. So far, the condition has been associated with about 20 genes at the genetic level, and further genetic variables are expected to be found. Numerous molecular pathways, including oligodendrocyte dysfunction, excitotoxicity, neuroinflammation, mitochondrial dysfunction and oxidative stress, cytoskeletal disruptions, and axonal transport defects, disrupted RNA metabolism, nucleocytoplasmic transport deficits, and compromised DNA repair, have been linked to the pathogenesis of ALS. It is interesting to note that many genes linked to ALS seem to be grouped along essential pathways, such as RNA metabolism, cytoskeletal and axonal transport, and protein quality control and disintegration [6].

Clinical features

After decades of research, ALS has been reclassified as a complicated condition with considerable variability in the clinical presentation, distribution of upper and lower neuron motor symptoms, and the location of disease origin.

Motor symptoms

Pathology of both the upper motor neuron (UMN) and lower motor neuron (LMN) is present in classical bulbar onset. Usually, dysphagia and dysarthria are the first symptoms, and then it progressively spreads to the limbs. The UMN is the main population affected by pseudobulbar palsy. It has a greater frequency in females, a longer survival time, and a pseudobulbar effect. It is marked by pronounced bulbar characteristics that gradually spread to the limbs. The disorder known as progressive bulbar palsy is characterized by pathology in the bulbar area and primarily affects the LMN.

Multiple clinical patterns of ALS exist, each with unique features. UMN and LMN diseases coexist in classical cervical onset ALS, which usually begins with hand weakness and progresses to the bulbar and lumbar areas. On the other hand, classical lumbar onset ALS begins with a foot drop and progresses to the cervical and bulbar regions over time. Flail arm ALS primarily affects men and progresses more slowly. It causes symmetrical proximal upper limb weakness and exhibits LMN involvement in the upper limbs and UMN involvement in the lower limbs. Symmetric lower limb weakness is the primary outcome of flail leg ALS, which is predominantly caused by LMN disease.

The condition known as primary lateral sclerosis (PLS) mainly affects the UMN but can start anywhere. If LMN symptoms appear within 4.5 years, PLS has the potential to progress to ALS. PLS is associated with an average life expectancy; however, it is essential to distinguish it from hereditary spastic paraplegia (HSP), especially if the affected limbs are symmetrical. Progressive muscular atrophy (PMA) primarily affects LMN and can start in any region; however, ALS is diagnosed if UMN symptoms appear within 4.5 years.

In respiratory onset ALS, both the UMN and LMN are involved, which usually manifests as respiratory problems before limb paralysis and has a worse survival rate. Predominantly affecting the distal LMN, pseudo-polyneuritis ALS first manifests as distal symptoms before possibly affecting the UMN. Monocular UMN involvement, which can develop into LMN symptoms, is a hallmark of hemiplegic ALS. Finally, cachexia is defined as unexplained weight loss, including muscular weakness and atrophy, that occurs before the characteristic presentation of ALS.

Cognitive and behavioral functions

Changes in behavior and cognition are common characteristics of many neurological disorders, such as FTD. A deficit in working memory can affect short-term memory and cognitive abilities as it is necessary for storing, retrieving, and modifying information in conscious awareness. Deficits in inhibition can cause impulsivity and an inability to disregard impulses, which makes it challenging to regulate how one responds to outside stimuli. Set shifting issues lead to inflexible thinking, trouble multitasking, and trouble adjusting to changing conditions. A person with impaired fluency may have disordered ideas and have trouble starting activities, which can hinder communication and task beginning.

Deficits in language function include problems with word identification, spelling, and grammar, which impact both expressive and receptive language skills. Passivity, a lack of initiative, and a decline in interest in once-enjoyable activities are characteristics of apathy. Impulsivity, a lack of self-control, improper social behaviors, impatience, and modifications in social conduct are all signs of disinhibition. Emotional detachment and decreased interpersonal warmth result from losing compassion or empathy.

Ritualistic actions, stereotypical speech patterns, and repetitive movements are examples of obsessive-compulsive, stereotyped, or persevering behaviors. Nutrition and general health may be impacted by changes in eating habits, including changed food choices, increased cigarette use, binge eating, hyperorality, and exploration of non-edible objects. Understanding and treating FTD and associated diseases requires an awareness of these cognitive and behavioral changes [3,5-7].

Treatment

Masitinib

Among ALS medications, masitinib stands out as a highly selective tyrosine kinase inhibitor that targets innate immune cells in the central and peripheral neurological systems, such as mast cell activity, macrophages, and microglia. It exhibited anti-tumoral, neuroprotective, and anti-inflammatory properties and was taken orally. Blocking vital growth and activation signaling pathways regulates the survival, migration, and degranulation of mast cells, indirectly regulating the range of proinflammatory and vasoactive mediators these cells may produce [1].

Stem Cell Therapeutic Approaches

Because cell-based treatments can target many pathogenic processes and replace lost or damaged cells, they have received a great deal of attention as a possible therapeutic strategy for ALS. Aspiration from the posterior iliac crest was used to retrieve bone marrow per standard protocol [8].

*Gene Therapy* 

Heritable gene expression changes that occur without DNA mutations are called epigenetics. Histone post-translational modifications (PTMs), microRNAs (miRNA), and DNA methylation are the three primary epigenetic processes [9].

Anti-sense oligonucleotides in familial amyotrophic lateral sclerosis (fALS): In the brain and spinal cord of SOD1-rats, it was shown that intracerebroventricular injections of ASO against SOD1 directly decreased SOD1 mRNA through RNase H activity, extending the rats' mean lifetime by 10 days. ASO administered intrathecally in humans was well tolerated and did not result in any significant side effects, according to the findings of a small number of experiments [4,10,11].

RNA interference in FALS: These double-stranded RNA duplexes, which range in length from 19 to 23 nucleotides, are broken down by the cell and put together to form an RNA-induced silencing complex (RISC), which targets mRNA in the cell. Like ASOs, RNAi affect gene expression in ways that support transcriptional silence, alternative splicing, and mRNA degradation [4,12,13].

Lentivirus (LV)-mediated gene therapy: Because of its broad cloning capability, natural ability to infect intact nuclear membranes, and capacity to transduce both dividing and non-dividing cells (e.g., neurons), LV was thought to be the best vector for gene therapy [4,14-16].

Lithium Carbonate

Lithium carbonate may slow the progression of UNC13A-ALS disease, though the exact mechanism is still unknown. Pre-clinical research indicates that lithium causes pyramidal neuron sprouting and synaptogenesis, which may counteract the previously reported decrease in synaptic transmission brought on by UNC13A depletion. Additional suggested pathways concern the control of intracellular calcium homeostasis and an increase in autophagy, a process that cells use to break down intracellular components and has been demonstrated to be triggered by lithium [17-19].

miRNA

miRNAs are an epigenetic mechanism that attach to Argonaute 2 and form the RISC, which lowers the expression of genes. Also, if there is a strong match between the mRNA and miRNA, the complex binds the 3'-UTR of that particular mRNA and destroys it [20,21].

Histone Phosphorylation and Ubiquitylation

Histone phosphorylation on serine, threonine, and tyrosine residues is crucial for gene expression, cell cycle progression, and DNA damage repair. Numerous enzymes involved in histone phosphorylation have been linked to ALS and other neurological conditions [22,23].

Ketogenic Diet

There has been increasing evidence that ketones protect neurons through various mitochondrial processes, such as elevated UCPs, improved antioxidant activity (lower reactive oxygen species (ROS) generation), and ATP synthesis. The primary ways that a ketogenic diet (KD) reduces ROS, boosts antioxidant levels, and regulates the activity of mitochondrial respiratory complexes are how it has an antioxidant effect on mitochondria. Rats given KD had higher glutathione levels in their hippocampal mitochondria and more active antioxidant enzymes in their hippocampal regions. According to recent research, one factor contributing to KD’s neuroprotective effects may be its ability to lower inflammatory reactions. Potential pathways for KD-based therapy that target the gut microbiota in neurological illness clinical trials might open up new therapeutic options. Moreover, it is feasible to deduce how KD is protective in various CNS illnesses by modifying the microbiome's composition [24-26].

Riluzole

The FDA authorized riluzole, an anti-glutamate agent created by Sanofi, as the first medication to manage ALS in 1995. By blocking glutamate presynaptic release and protein kinase C, deactivating voltage-dependent sodium channels, slowly inactivating potassium channels, and limiting glutamate presynaptic release, it can prevent MNs from overexciting and reduce excitotoxic neuronal cell death. The most common side effects include transiently raised liver enzyme levels, nausea, and weakness [27,28].

*Edaravone* 

Edaravone can lessen oxidative stress since it is a free radical scavenger. By acting as a scavenger of free radicals, edaravone helps protect the neurological system from the harmful consequences of oxidative stress. Oxidative stress is a significant factor in the degradation of MNs in ALS, which results in motor function loss and muscular weakening. Edaravone helps prevent oxidative damage and eventually preserves MN function by reducing the amount of harmful ROS generated by MNs. Furthermore, edaravone possesses anti-inflammatory properties, which may be crucial for ALS patients, given that the illness is partly caused by neuroinflammation. Edaravone may assist people with ALS in delaying the loss of motor function by decreasing inflammation. Administered intravenously, edaravone is usually given in a cyclical manner consisting of daily infusions for a predetermined number of days, interspersed by breaks and repeat cycles. It is not a cure for ALS; its usage is intended to preserve motor function and halt the disease's development. Edaravone's benefits are more pronounced in the early stages of the disease, and it is especially recommended for ALS patients who still have some respiratory function [29].

Anti-apoptotic Drug

AMXOO35, a combination of sodium phenylbutyrate and taurursodiol (tauroursodeoxycholic acid), aims to reduce neuronal death by simultaneously targeting the mitochondria and endoplasmic reticulum (ER). It inhibits the dual apoptosis of UPR-Bax [29,30]. The regenerating of methionine from homocysteine is carried out by methionine synthase, which is a cofactor of methylcobalamin, the active form of vitamin B12. It can aid in removing neurotoxic homocysteine, which can cause oxidative stress, inflammation, and cellular dysfunction, ultimately resulting in MN injury and cell death [29,31]. By inhibiting the DLK/c-Jun N-terminal kinase pathway, GDC-0134, a dual leucine zipper kinase (DLK) inhibitor, can prevent axon degeneration and neuronal apoptosis [29].

Anti-inflammatory and Anti-excitotoxic Drugs

Myeloperoxidase (MPO), a pro-oxidant enzyme found in triggered macrophages and microglia, is inhibited by verdiperstat. Reduced inflammation and oxidative stress levels may result from MPO suppression [32]. Recombinant IL-2 Proleukin (Aldesleukin) can stimulate Treg proliferation and improve Treg function. For Tregs to develop, get activated, and survive, IL-2 is an essential cytokine [29]. Another sodium channel blocker that is utilized is mexiletine.

Assistive treatment and rehabilitation

For people with ALS to preserve their freedom, capacity for communication, and general quality of life, assistive technology is crucial. The choice of particular devices is contingent upon the requirements of each patient and the course of the illness; identifying the best options frequently necessitates consultation with healthcare professionals and assistive technology experts. For those suffering from ALS, assistive technology is essential to preserving their independence and quality of life. As ALS gradually compromises motor function, patients find it challenging to carry out daily duties. The use of assistive technology aids in making up for these physical constraints. ALS patients employ the following kinds of assistive technology.

Due to gradual diaphragm weakening, chronic neuromuscular respiratory failure is the most prevalent cause of morbidity and death in people with ALS. It has been demonstrated that non-invasive breathing increases ALS patients' life expectancy by an average of 205 days [33]. As ALS progresses, neuromuscular respiratory weakness impairs secretion clearance and coughing. It also increases the risk of pneumonia, which raises morbidity and death. Mucus-mobilizing and aided coughing techniques are two often advised methods to help in clearing up secretions [34].

For 20% of people with ALS who have bulbar onset, dysarthria may be the presenting symptom. The main strategies for treating dysarthria in ALS patients focus on teaching the patient and their family how to communicate with aids and augmentative devices. Patients who have functional limbs at the beginning of the disease may benefit from low-tech choices like a writing board. Text-to-speech applications, which allow patients to enter or choose words and phrases on their smartphone screen, are among the most widely used aids. Patients can employ eye gazing software, which detects eye movements to operate a cursor on the screen if their motor function prevents them from operating a device with their limbs [35,36].

Patients with spasticity experience morbidity. However, baclofen is the most often prescribed antispasticity drug in ALS multidisciplinary clinics. For ALS, the greatest dosages are seldom required [37]. The starting dose is 5 mg to 10 mg, given two to three times a day, and can be gradually increased to 20 mg, given four times daily.

Mobility assistance such as power wheelchairs: For those unable to walk, these motorized wheelchairs with sophisticated controls, including joysticks or head array systems, offer mobility. Movement scooters can improve movement, particularly in more significant indoor and outdoor locations. They come in a variety of sizes and functions. Systems for mounting wheelchairs: With the help of these solutions, users may easily attach computer tablets or communication devices to their wheelchairs.

Users of environmental control systems can regulate thermostats, lights, appliances, and other items in their surroundings. Standard interfaces include switches, voice commands, and smartphone apps.

Adaptive computer technology, such as head- or eye-tracking systems, allows users to move a computer's cursor with their head or eyes, facilitating software use, online browsing, and communication. Customized mice and keyboards: Typing and navigating are more straightforward with specialized keyboards and mice made for those with poor dexterity or muscular power. Accessibility for those utilizing wheelchairs or other mobility aids is improved by home modifications such as ramps and lifts. Bathroom modifications: An accessible bathroom with amenities like grab bars and roll-in showers is crucial for independent living.

Assistive devices for daily living include adaptive utensils, buttoning aids, and dressing sticks, which help with daily activities such as eating and dressing. Orthoses and braces like custom-made orthoses and braces can help maintain joint function and reduce the risk of contractures [37].

Recent pharmaceutical advancement 

Rapamycin

**Figure 2:**
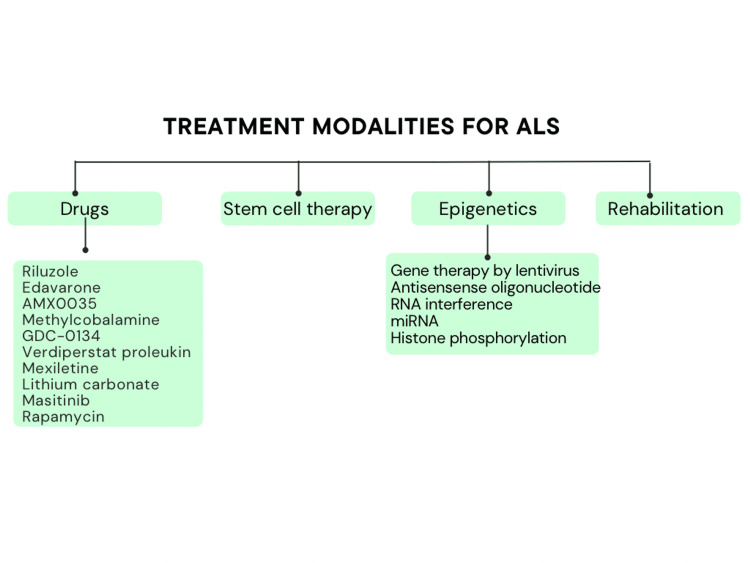
Newer modalities and treatments of amyotrophic lateral sclerosis miRNA: Microribonucleic acid; RNA: ribonucleic acid

In a trial, rapamycin was shown to affect neuroinflammation by influencing autophagy and growing regulatory T cells, two key components in the pathophysiology of ALS. It raised the proportion of B cells and monocytes while lowering IL-18 protein and mRNA relative expression of the pro-inflammatory cytokine IL-18 [38]. Figure 2 depicts newer treatment modalities for ALS.

Table 1 depicts the reference summary table.

**Table 1:** Summary of the articles included in the review ALS: Amyotrophic lateral sclerosis; DNA: deoxyribonucleic acid; RNA: ribonucleic acid; fALS: familial amyotrophic lateral sclerosis; FDA: Food and Drug Administration; SOD: superoxide dismutase; ASO: antisense oligonucleotides; MPO: myeloperoxidase

Sr No.	Author	Article type	Year	Interpretation
1	Ketabforoush, et al., [1]	Review article	2023	Masetinib, which targets immune cells in the neurological system such as mast cells, macrophages, and microglia. It regulates mast cell activity and lowers inflammation while having antitumoral, neuroprotective, and anti-inflammatory properties.
2	Alonso et al., [2]	population-based study	2009	Most instances of ALS occur in the 40–70 age range, peaking in the 58–63 range for sporadic forms and 47–52 for familial forms.
3	Logroscino et al., [3]	Review Article	2019	Motor neurons are impacted by ALS (Amyotrophic Lateral Sclerosis), which frequently manifests randomly. In most situations, TDP43 protein aggregates occur, with the exception of rare hereditary alterations.
4	Cappella et al., [4]	Review article	2019	The main cause of death is respiratory failure, occurring approximately 3–5 years after diagnosis.
5	Chiò et al., [5]	Critical review	2009	Respiratory failure is the primary cause of mortality, which develops three to five years following diagnosis.
6	Masrori et al., [6]	Clinical review	2020	A combination of aging-related malfunction, environmental variables, and hereditary factors results in ALS.
7	Goutman et al., [7]	Clinical review	2022	clinical features of ALS.
8	Mazzini et al., [8]	Clinical trial	2008	Cell-based therapies are promising for ALS, as they can address various disease factors and replace damaged cells. Bone marrow is collected from the iliac crest as a standard procedure.
9	Allis et al., [9]	Clinical review	2016	Heritable modifications in gene expression without DNA mutations are the subject of epigenetics. Among the crucial processes are DNA methylation, microRNAs, and histone changes.
10	Miller et al., [10]	Clinical trial	2013	adASO administered intrathecally to humans was well tolerated and did not result in any significant adverse effects.
11	Smith et al., [11]	Clinical trial	2006	Through RNase H activity, intracerebroventricular injections of ASO against SOD1 directly reduced SOD1 mRNA.
12	Meister et al., [12]	Clinical review	2004	RNA interference in fals.
13	Hannon et al., [13]	Clinical review	2004	RNA interference in fals.
14	Armandola et al., [14]	Clinical review	2004	Lentivirus-mediated gene therapy
15	Thomas et al., [15]	Clinical review	2003	Lentivirus-mediated gene therapy
16	Azzouz et al., [16]	Clinical review	2006	Lentivirus-mediated gene therapy
17	Willemse et al., [17]	Clinical review	2023	The development of UNC13A-ALS disease may be slowed down by lithium carbonate.
18	Pasquali et al., [18]	Clinical review	2009	The development of UNC13A-ALS disease may be slowed down by lithium carbonate.
19	Limanaqi et al., [19]	Clinical review	2019	The development of UNC13A-ALS disease may be slowed down by lithium carbonate.
20	Tolia et al., [20]	Clinical review	2007	miRNAs are an epigenetic mechanism that attach to Argonaute 2 and form the RNA-induced silencing complex, which lowers the expression of genes.
21	Tétreault et al., [21]	Clinical review	2013	miRNAs are an epigenetic mechanism that attach to Argonaute 2 and form the RNA-induced silencing complex (RISC), which lowers the expression of genes.
22	Bennett et al., [22]	Clinical review	2019	Histone phosphorylation and ubiquitylation as a treatment option of ALS.
23	Sawicka et al., [23]	Clinical review	2012	Histone phosphorylation and ubiquitylation as a treatment option of ALS.
24	Gough et al., [24]	Clinical review	2021	There is mounting evidence that ketones protect the brain via a variety of mitochondrial processes.
25	Jarrett et al., [25]	Clinical trial	2008	There is mounting evidence that ketones protect the brain via a variety of mitochondrial processes.
26	Ziegler et al., [26]	Clinical trial	2003	There is mounting evidence that ketones, via a variety of mitochondrial processes, protect the brain.
27	Xu et al., [27]	Clinical trial	2001	The FDA authorized riluzole, an anti-glutamate agent created by Sanofi, as the first medication for the management of ALS.
28	Benoit et al., [28]	Clinical trial	1991	The first drug approved by the FDA to treat ALS is the anti-glutamate compound riluzole, which was developed by Sanofi.
29	Jiang et al., [29]	Review article	2022	Edavarone can lessen oxidative stress since it is a free radical scavenger.
30	Paganoni et al., [30]	Clinical trial	2020	By simultaneously targeting the mitochondria and endoplasmic reticulum (ER), AMX0035 aims to reduce neuronal death.
31	Zoccolella et al., [31]	Clinical trial	2010	methylcobalamin, the active form of vitamin B12 used in the treatment of ALS. It can aid in the removal of neurotoxic homocysteine, which can cause oxidative stress, inflammation, and cellular dysfunction, ultimately resulting in motor neuron injury and cell death
32	Stefanova et al., [32]	Clinical trial	2012	Verdiperstat inhibits myeloperoxidase (MPO), a pro-oxidant enzyme present in activated macrophages and microglia. MPO inhibition may lead to decreased oxidative stress and inflammation levels.
33	Bourke et al., [33]	Clinical trial	2006	It has been demonstrated that non-invasive breathing increases the average survival time of ALS patients by 205 days.
34	Lechtzin et al., [34]	Cohort study	2016	It is often advised to use aided coughing and mucus-mobilizing strategies to help with secretion clearance.
35	Beukelman et al., [35]	Review article	2011	Patients can employ eye gazing software, which detects eye movements to operate a cursor on the screen, if their motor function prevents them from operating a device with their limbs.
36	Brownlee et al., [36]	Review article	2007	If a patient's motor function makes it impossible for them to move their limbs to control a device, they can use eye gazing software, which tracks eye movements to move a pointer on the screen.
37	Brent et al., [37]	Clinical review	2020	Orthoses and Braces like custom-made orthoses and braces can help maintain joint function and reduce the risk of contractures.
38	Mandrioli et al., [38]	Clinical trial	2023	Rapamycin was shown to affect neuroinflammation by influencing autophagy and growing regulatory T cells, two key components in the pathophysiology of ALS. It raised the proportion of B cells and monocytes while lowering IL-18 protein and mRNA relative expression of the pro-inflammatory cytokine IL-18.

## Conclusions

Finally, an overview of the intricate and debilitating neurodegenerative illness known as ALS has been given in this article. We talked about the clinical manifestations and pathogenesis, which helped clarify this illness's complex nature. We also discussed the new therapeutic approaches and treatments for ALS, emphasizing the continuous efforts in the area to discover efficient cures. Even with the significant advancements in care, ALS is still a problematic illness with few therapeutic choices. However, there is optimism for future discoveries due to the recent progress in understanding the disease's genetic and molecular foundations. To create new treatments that might lessen ALS patients' suffering and eventually lead to a cure, researchers, physicians, and pharmaceutical corporations must work together. We believe that this study will greatly assist researchers and clinicians in their quest for more potent therapies and a better understanding of ALS. We are getting closer to making ALS patients' lives better by continuing to investigate the complexities of this terrible illness.
